# Intermedin Attenuates LPS-induced Inflammation in the Rat Testis

**DOI:** 10.1371/journal.pone.0065278

**Published:** 2013-06-04

**Authors:** Lei Li, Ping Ma, Yongjun Liu, Chen Huang, Wai-sum O, Fai Tang, Jian V. Zhang

**Affiliations:** 1 Research Center for Gene and Cell Engineering, Institute of Biomedicine and Biotechnology, Shenzhen Institute of Advance Technology, Chinese Academy of Sciences, Shenzhen City, China; 2 Department of Anatomy, Li Ka Shing Faculty of Medicine, University of Hong Kong, Hong Kong, China; 3 Department of Physiology, Li Ka Shing Faculty of Medicine, University of Hong Kong, Hong Kong, China; 4 Shenzhen Engineering Laboratory of Single-Molecule Detection and Instrument Development, Shenzhen City, China; 5 Innovative Pharmacology and Biotherapy Pre-Clinical Test Public Service Platform of Shenzhen, Shenzhen City, China; University of Kansas Medical Center, United States of America

## Abstract

First reported as a vasoactive peptide in the cardiovascular system, intermedin (IMD), also known as adrenomedullin 2 (ADM2), is a hormone with multiple potent roles, including its antioxidant action on the pulmonary, central nervous, cardiovascular and renal systems. Though IMD may play certain roles in trophoblast cell invasion, early embryonic development and cumulus cell-oocyte interaction, the role of IMD in the male reproductive system has yet to be investigated. This paper reports our findings on the gene expression of IMD, its receptor components and its protein localization in the testes. In a rat model, bacterial lippolysaccharide (LPS) induced atypical orchitis, and LPS treatment upregulated the expression of IMD and one of its receptor component proteins, i.e. receptor activity modifying protein 2 (RAMP2). IMD decreased both plasma and testicular levels of reactive oxygen species (ROS) production, attenuated the increase in the gene expression of the proinflammatory cytokines tumor necrosis factor alpha (TNFα), interleukin 6 (IL6) and interleukin 1 beta (IL1β), rescued spermatogenesis, and prevented the decrease in plasma testosterone levels caused by LPS. The restorative effect of IMD on steroidogenesis was also observed in hydrogen peroxide-treated rat primary Leydig cells culture. Our results indicate IMD plays an important protective role in spermatogenesis and steroidogenesis, suggesting therapeutic potential for IMD in pathological conditions such as orchitis.

## Introduction

Male factor infertility accounts for up to 50% of all cases of infertility and affects one out of twenty men [1]. Evidence shows oxidative stress is a common factor in testicular dysfunction, inhibiting both Leydig cell steroidogenesis and spermatogenesis [2], [3]. Oxidative stress results when reactive oxygen species (ROS) produced by the oxidation of lipids in membranes, amino acids in proteins, and carbohydrates within nucleic acids are in excess. Among the factors that cause oxidative stress in the testes are radiation, cryptorchidism, testicular torsion and diabetes [2], [3]. Oxidative stress from orchitis/testicular infection may have transient or even permanent effects on male fertility [2], [3]. In an experimental model involving the intraperitoneal injection of bacterial lipopolysaccharide (LPS), lipid peroxidation was induced in the testes accompanied by a significant decrease in testosterone production and disruption of spermatogenesis [4], [5].

Intermedin (IMD), also known as adrenomedullin 2 (ADM2), is a newly discovered hormone that belongs to the calcitonin, amylin, calcitonin gene-related peptides (CGRP) and adrenomedullin (ADM) peptide family. It was independently cloned by two groups [6], [7] and was shown to be expressed in the vasculature and a variety of tissues in other organs including the stomach, kidney, pituitary and placenta [6], [7]. Like CGRP and ADM, IMD uses the common receptor complexes which consist of calcitonin receptor-like receptor (CLR) and one of the three receptor activity-modifying proteins (RAMP1, RAMP2 and RAMP3) [6], [7]. Some of the reported actions of IMD are similar to those of ADM and CGRP, and most of its roles are found in the cardiovascular system. For example, intravenously IMD injection lowered blood pressure [1], [6], [7], [8] and intracerebroventricular injection of IMD elevated blood pressure and heart rate [8].

IMD is noted for its protective role in tissue injury, such as injuries to the central nervous, pulmonary, cardiovascular and renal systems. IMD has been shown to protect rat cerebral endothelial cells from oxidative damage in vitro [9] and attenuate ventilator-induced lung injury in mice [10]. The increase in myocardial oxidative stress was accompanied by a robust augmentation of IMD expression in hypertrophied left ventricular cardiomyocytes of spontaneously hypertensive rats (SHRs), compared with normotensive Wistar–Kyoto rats [11]. IMD attenuated oxidative stress injuries in the myocardium in a rat ischemia/reperfusion model [12] and also in the blood vessels and kidneys of DOCA-salt hypertensive rats [13].

The expression of IMD has been reported in trophoblast cells in human placenta [14], [15]. The antagonism of IMD function in pregnant rats demonstrated the important role of IMD during implantation and early embryonic development [16], which was further supported by the association of lower expression of IMD in serum, villi and decidua with first-trimester spontaneous abortion in patients [17]. Recently, IMD produced by the oocyte has proven to be important for the regulation of cell interactions in cumulus cell-enclosed oocyte complexes partly through its suppressive action on cumulus cell apoptosis [18]. In contrast to the research on the effects of IMD in the female reproductive system, no biological function of IMD in the testes has been reported.

All in all, it would be interesting to know whether or not IMD and its receptors are expressed in the testes and whether it plays a similar protective role in testicular oxidative stress. This study was undertaken to investigate the possible expression of IMD in the testes and its potential roles in testicular oxidative stress in a rat model of LPS-induced atypical orchitis.

## Materials and Methods

### Ethics Statement

Normal human testes paraffin sections were purchased from Pantomics (Pantomics, CA, USA: cat.no.TES01). Three-month-old Balb/c mice and Sprague-Dawley rats were obtained from the Laboratory Animal Center, Institutes of Biomedicine and Health, Chinese Academy of Sciences, China. The animals were housed at a constant temperature and humidity, with a 12-hour light-dark cycle. Chow and water were available ad libitum. All procedures related to animal usage were approved by the Committee on the Use of Live Animals for Teaching and Research, Shenzhen Institutes of Advanced Technology, Chinese Academy of Sciences (Permit Number: SIAT-IRB-120223-A0009).

### IMD Tissue Extraction and Measurement by Radioimmunoassay (RIA)

Tissues, including the testes, epididymis, vas deferens, seminal vesicle, coagulating gland and prostate were harvested from three-month-old rats immediately after decapitation. Tissues were homogenized in 2N ice-cold acetic acid and then boiled for 10 min. A 50 µl aliquot was taken for Bio-Rad protein assay (Bio-Rad Laboratories, CA, USA: cat.no.500-0201EDU). The remaining homogenates were centrifuged at 18, 600 g for 20 min at 4°C. The supernatants were lyophilized and reconstituted in 1 X RIA assay buffer. IMD peptide concentrations were measured in duplicate using an Intermedin (Rat) - RIA Kit (Phoenix Pharmaceuticals, CA, USA: cat.no.RK-010-52). The minimum detectable concentration was 76 pg/ml and the range was 20–2, 560 pg/ml.

### LPS and/or IMD Treatment and Tissue Collection

The rats were divided into three groups. While under anesthesia, one group were intraperitoneally injected with saline (0.2 ml/rat) only, one group with saline containing 4 mg/kg body weight (BW) LPS (E.coli serotype 026:B6 from Sigma, MO, USA: cat.no.L8274) and the third group with 4 mg/kg BW LPS injection, followed by right femoral vein infusion of 10 nmol/kg BW of rat IMD (Scipeptide, Shanghai, China: cat.no.P011090601) for 10 min. At 6 h, 12 h and 72 h after LPS injection, 6–7 rats from each group were weighed and decapitated. Blood was collected and centrifuged at 1, 200 g for 10 min. The plasma was measured for testosterone by RIA and its lipid peroxidation level assessed by TBARS assay. One testis from each rat was decapsulated and stored at -80°C for further analysis of gene expression or testicular lipid peroxidation. The other testis was fixed in 4% formaldehyde together with its epididymis for histological studies.

### Primary Leydig Cell Culture

Leydig cells were isolated from testes of 3-month-old male rats and were cultured for 2 days as described previously with some modifications [19]. Five rats were killed by decapitation. The testes were excised rapidly and washed twice in 1× PBS. The decapsulated testes were digested for 15 min in a shaking water-bath at 80 cycles/min at 34°C in a flask containing DMEM/F12-0.1% BSA (Sigma, MO, USA: cat.no.A7906) supplemented with 0.5 mg/ml collagenase (Sigma, MO, USA: cat.no.C0130) and 0.25 mg/ml soybean trypsin inhibitor (Sigma, MO, USA: cat.no.T6522). Ice-cold medium was added to the flask to stop the digestion. The suspension was allowed to settle for 5 min, and the supernatant containing Leydig cells was filtered through cell strainers (70 µm nylon, Falcon BD Biosciences, Germany: cat.no.352350). The tubules were dispersed in another 50 ml medium, and the supernatant was pooled and centrifuged. The Leydig cells were separated by discontinuous Percoll (Amersham Biosciences, Uppsala, Sweden: cat.no.17-5445-01) gradients (with six density fractions ranging from 1.030 to 1.096 g/ml). Leydig cells located at the boundary between fractions of 1.070 and 1.096 g/ml densities were collected and washed twice with ice-cold DMEM/F12-0.1%BSA medium. The collected Leydig cells were seeded in a NUNC 24-multiwell plate (NUNC, Roskilde, Denmark: cat.no.142475). The cells were pre-incubated in DMEM/F12-0.1%BSA at 34°C in a humidified atmosphere of 5% CO2/95% air. After 24 h incubation, the cell purity was determined by 3β-hydroxysteroid dehydrogenase (3β-HSD) staining. The Leydig cells were treated for 24 h with 250 µM H_2_O_2_, 100 nM rat IMD or a combination of H_2_O_2_ and IMD or culture media only. The media were collected for testosterone measurement by RIA.

### RNA Analysis by QPCR

The total RNA of the tissues and cells was extracted using TRIZOL reagent (Invitrogen Life Technologies; cat.no. 15596-018) and subjected to QPCR analysis. RNA samples (1 µg) were reverse transcribed into cDNA according to the manufacturer’s instructions (Bio-Rad Laboratories, CA, USA: cat.no.170-8890). The PCR reaction mixtures contained 10 µl SYBR Premix Ex TaqTM II (Takara, Japan: cat.no.DRR820), 500 nM of each primer, 1 µl template cDNA, and DNase-free water to a final volume of 20 µl. Cycle conditions were 95°C for 10 sec, followed by 45 cycles of 95°C for 5 sec, 60°C for 30 sec, and 72°C for 30 sec. The reaction was completed with a dissociation step for melting point analysis from 50°C to 95°C (in increments of 0.5°C for 10 sec each). The primers were designed on the basis of the published sequences of IMD (GCTGATGGTCACGGTAAC, forward; CGCTGGAAGGAATCTTGG, reverse; NM_201426.1), CLR (CCAAACAGACT TGGGAGTCACTAGG, forward; GCTGTCTTCTCTTTCTCATGCGTGC, reverse; NM_012717.1); RAMP1 (CACTCACTGCACCAAACTCGTG, forward; CAGTCATGAGCAGTGTGACCGTAA, reverse; NM_031645.1); RAMP2 (AGGTATTACAGCAACCTGCGGT, forward; ACATCCTCTGGGGGATCGGAGA, reverse; NM_031646.1); RAMP3 (ACCTGTCGGAGTTCATCGTG, forward; ACTTCATCCGGGGGGTCTTC, reverse; NM_020100.2) and tumor necrosis factor alpha (TNFα) (AAATGGGCTCCCTCTCATCAGTTC, forward; TCTGCTTGGTGGTTTG CTACGAC, reverse; NM_012675.3), interleukin 6 (IL6) (TCCTACCCCAACTTCCAATG CTC, forward; TTGGATGGTCTTGGTCCTTAGCC, reverse; NM_012589.1), interleukin 1 beta (IL1β) (CATTGTGGCTGTGGAGAAG, forward; ATCATCCCACGAGTCACAGA, reverse; NM_031512.2), and beta-actin (GGAAATCGTGCGTGACATTA, forward; AGGAAGGAAGGCTGGAAGAG, reverse; NM_031144.3) for rats. The relative gene expression levels were normalized to beta-actin using the^ ΔΔ^CT method, where CT was the cycle threshold. Melt curve analysis for each primer set revealed only one peak for each product.

### Histological Studies and Immunocytochemistry

The testes and epididymis were fixed and processed for embedding in paraffin, sectioned (5 µm), and stained for histological analysis by hematoxylin and eosin. Immunohistochemistry of the testes was performed on 5 µm sections of paraffin-embedded tissues with a peroxidase-labeling kit (Vector Laboratories, CA, USA: cat.no.PI-9500) using a rabbit anti-rat intermedin antibody (Phoenix Pharmaceuticals, CA, USA: cat.no.H-010-52). Staining was visualized using a DAB substrate kit for peroxidase (Vector Laboratories, CA, USA: cat.no.SK-4105) counterstained with hematoxylin. The primary antibody was omitted from control sections to check for nonspecific staining.

### Testosterone Measurement by RIA

The testosterone levels in the plasma and culture media were measured using a commercial Iodine^ [125I]^ Radioimmunoassay Kit (Lareneen, Guangzhou, China: cat.no.S-004). The sensitivity of the testosterone RIA assay was 0.2 ng/dL, and the intra-assay and inter-assay errors were less than 10% and 15% respectively.

### Lipid Peroxidation Assessment by TBARS Assay

The levels of lipid peroxidation in the plasma and testes homogenate were measured as TBARS using an oxiSelect TBARS assay kit (MDA Quantitation, Cell Biolabs, CA, USA: cat.no.STA-330). Plasma samples were assayed directly using the kit. The testes were first homogenized on ice at 100 mg/ml in PBS containing 1X butylated hydroxytoluene (BHT) provided in the kit and then centrifuged at 10, 000 g for 5 min to collect the supernatant. The TBARS assay was used on 100 ul, while another 100 ul was measured for protein normalization (Bio-Rad Laboratories, CA, USA: cat.no.500-0201EDU).

### Statistical Analysis

All data were expressed as mean ± SEM, and statistical significance was assessed by one-way ANOVA followed by Student–Newmann–Keuls test or Student t-test. Statistical significance was taken at the P<0.05 level.

## Results

### The Expression of IMD and its Receptor Components in Human and Rodent Testes

**Figure 1:**
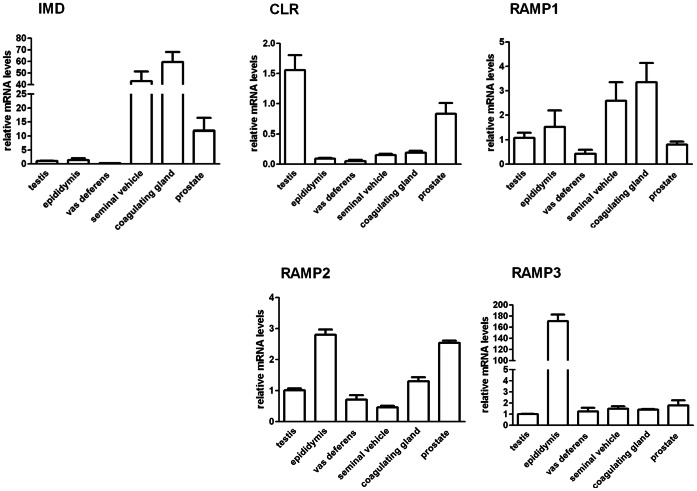
Gene expression of IMD and its receptor components in rat male reproductive systems. The relative gene expression of intermedin (IMD) and its receptors components CLR, RAMP1, RAMP2 and RAMP3 in adult rat male reproductive systems including testes, epididymis, vas deferens, seminal vesicles, coagulating glands and prostate was analyzed by QPCR. Beta-actin served as the reference gene; all data were expressed as mean ± SEM; n = 5–6.

**Figure 2:**
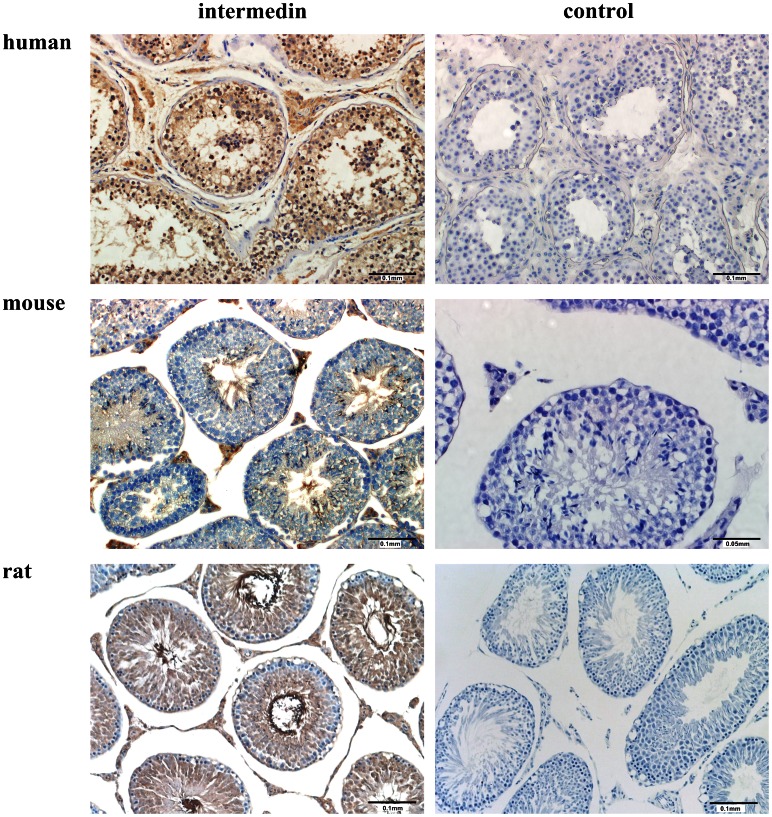
Immunolocalization of IMD in human, mouse and rat testis sections. Intermedin positive immunostaining was located to both in the interstitial Leydig cells and to a lesser extent in the seminiferous tubules, including the tails of the spermatids. Representative sections of n = 4 stain with similar results; negative control omitting primary antibody for each section was presented in parallel at right panel; scale bars, 0.1 mm and 0.05 mm as indicated in each picture.

QPCR analysis showed the gene expression of IMD and its receptor components in male reproductive system of rats, including the testes (FIG.1). Compared with seminal vehicles and coagulating glands, the gene expression of IMD is relatively low, while those of CLR and RAMP2 are relatively high in the testes. The epididymis also showed a relatively high expression level of RAMP2 and RAMP3. Immunohistochemistry localized IMD in human, mouse and rat testes, both in the interstitial Leydig cells and to a lesser extent in the seminiferous tubules, including the tails of the spermatids (FIG.2). RIA analysis showed that the testes had the highest level of IMD peptide (113.1±19.5 fmol/mg protein) in rats compared with those in the accessory sex glands (Table 1). The high level of IMD and the expression of its receptor components in the testes imply a potentially important biological role.

**Table 1:** IMD levels in rat male reproductive tract.

Tissues	IMD (fmol/mg protein)
Testis	113.1±19.5
Seminal vesicle	73.6±12.2
Coagulating gland	73.5±7.2
Prostate	45.3±6.4
Epididymis	55.6±3.0

### Effects of IMD on LPS-induced Changes in Body Weight, Plasma Testosterone Levels in Vivo and H_2_O_2_-induced Change in Testosterone Production in Vitro

**Figure 3:**
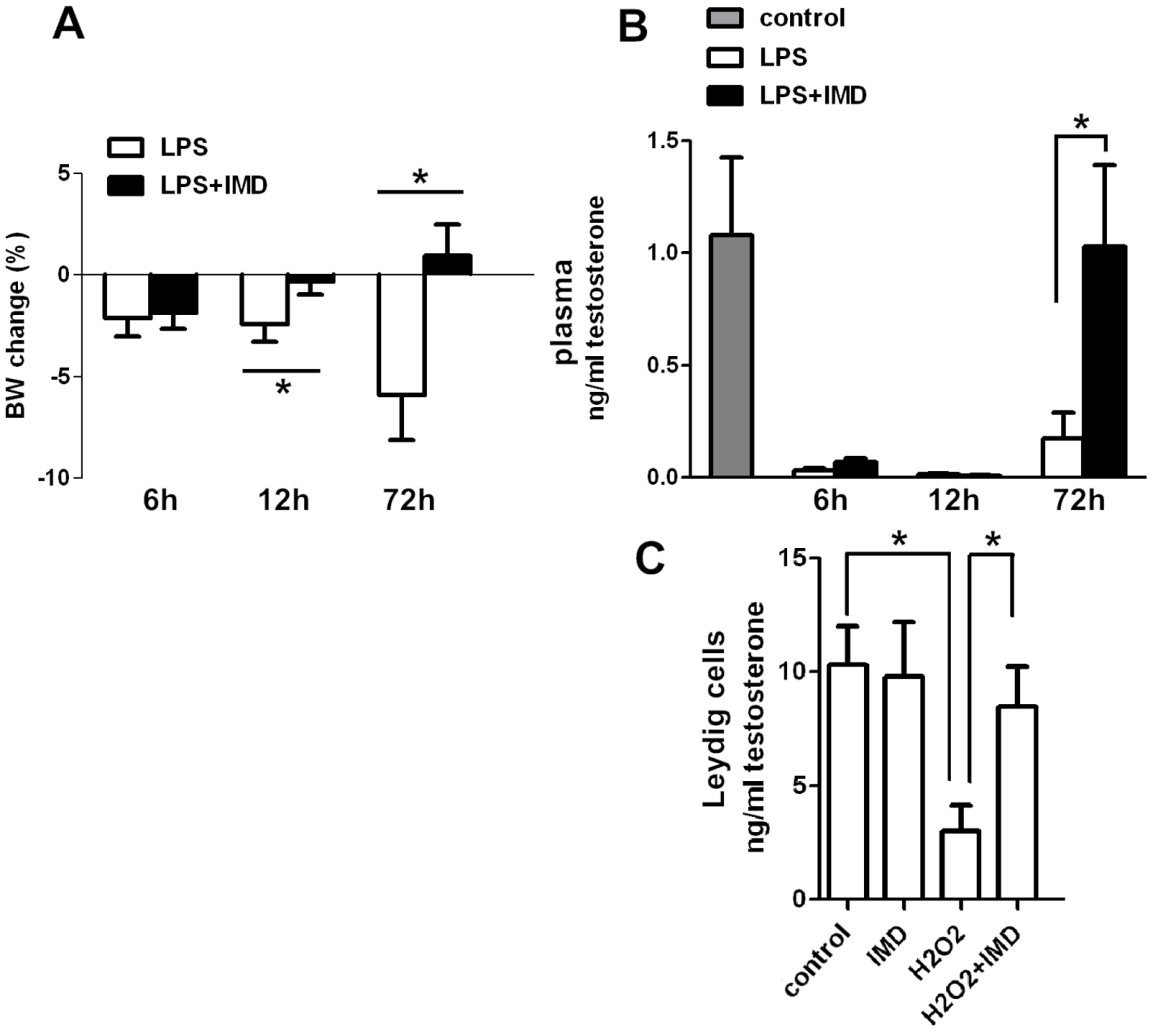
Effects of IMD on the LPS-induced changes in body weight, plasma testosterone levels in vivo and H_2_O_2_-induced change in testosterone production in vitro. IMD cotreatment restored the decreased body weight (A), plasma testosterone levels (B) 72 h after LPS treatment. IMD also rescued the H_2_O_2_ suppressed testosterone production from primary Leydig cells (C). All data were expressed as mean ± SEM; * P<0.05 by one-way ANOVA followed by Student–Newmann–Keuls test for (A) and (B); * P<0.05 by Student t-test for (C); n = 5–7.

Compared with saline control, the body weight (BW) of LPS-treated animals decreased at 6 h, 12 h and 72 h after LPS injection. Cotreatment of IMD with LPS attenuated this decrease in BW at 12 h and 72 h after treatment (FIG.3A). LPS also induced a dramatic drop in plasma testosterone levels, which persisted at 6 h, 12 h and 72 h after LPS treatment. IMD cotreatment restored the LPS-suppressed testosterone levels back to normal at 72 h (FIG.3B). The same effect of IMD on H_2_O_2_-suppressed testosterone production in vitro was demonstrated in primary Leydig cell cultures from rats (FIG.3C), though IMD showed no effect on basal testosterone production (data now shown).

### Effect of IMD on LPS-induced Histological Changes in the Testes

**Figure 4:**
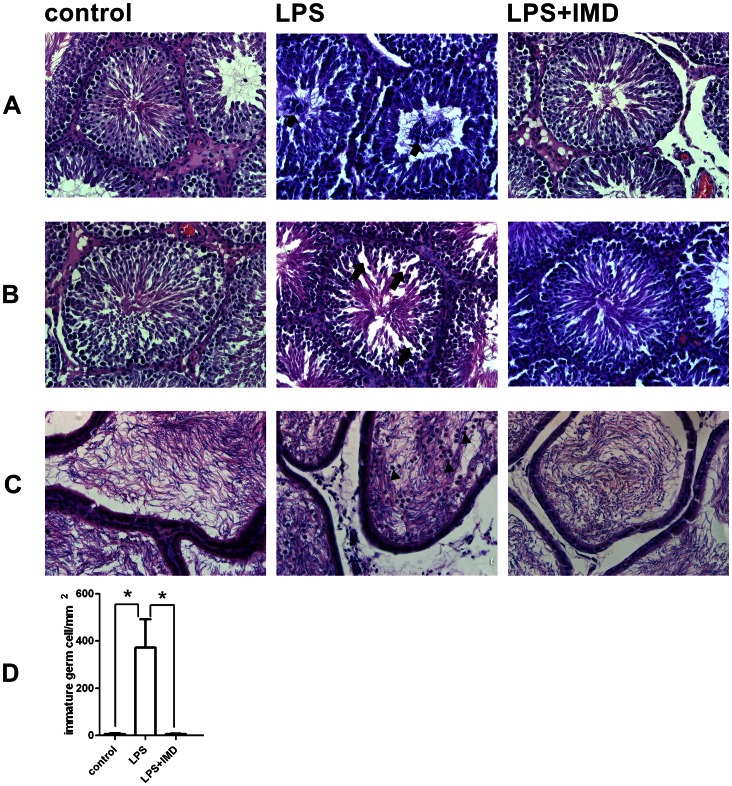
Histological examination of the rat testes and epididymis after LPS treatment or LPS and IMD cotreatment compared with saline control. (A) LPS-treated testes 6 h after injection, showing accumulation of immature germ cells (indicated by arrows) in the seminiferous tubule lumen, which was not observed in the IMD-cotreated testis. (B) LPS-treated testes 72 h after injection, showing increased inter cellular gaps (indicated by arrows) due to disruption of cell-cell contract and/or loss of cells in the seminiferous epithelium, which was not observed in the IMD-cotreated testis. (C) LPS-treated epididymis 72 h after injection, showing large numbers of round immature germ cells (indicated by arrowheads) in the epididymal lumen, which was not observed in the IMD-cotreated epididymis. (D) Quantitive presentation of the detached round immature germ cells in the epididymal lumen by counting five random fields under 20X magnification. Data were expressed as mean ± SEM; * P<0.05 by Student t-test for (D); For (A), (B), and (C), representative sections of n = 5–7 rats with similar results.

Histological examination of the testes and epididymis sections revealed the disruptive effects of LPS on testicular morphology. Compared with saline control, LPS-treated testes at 6 h after injection showed an accumulation of immature germ cells in the lumen (lumina) of some seminiferous tubules. Such an accumulation was not observed in the IMD-cotreated testes (FIG.4A). LPS-treated testes 72 h after injection showed an increase in inter-cellular gaps, due to the disruption of cell-cell contact or loss of cells in the seminiferous epithelium (FIG.4B), accompanied by large numbers of round, immature germ cells in the epididymal lumen (FIG.4C); this was not found with the IMD cotreatment (FIG.4B&C). Counting five random fields under 20X magnification showed a significant increase in the number of sloughing immature germ cells in LPS-treated epididymis compared with saline control, and this increase was eliminated with IMD cotreatment (FIG.4D).

### Effect of IMD on LPS-induced Plasma Lipid Peroxidation and Testicular Lipid Peroxidation

**Figure 5:**
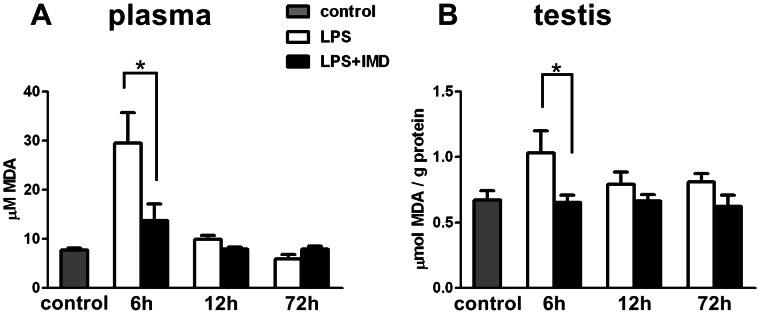
IMD cotreatment suppressed the LPS-induced lipid peroxidation in rats. Lipid peroxidation was assessed by TBARS assay (MDA quantitation) in plasma and in the testis 6 h, 12 h, 72 h after LPS treatment or LPS and IMD cotreatment compared with saline control. (A) Cotreatment of IMD suppressed the LPS-induced increase in circulating lipid peroxidation levels 6 h after treatment. (B) Cotreatment of IMD suppressed the LPS-induced increase in testicular lipid peroxidation levels 6 h after treatment. All data were expressed as mean ± SEM; * P<0.05 by one-way ANOVA followed by Student–Newmann–Keuls test; n = 5–7.

Lipid peroxidation levels in plasma and testicular tissues were evaluated by TBARS assay through MDA quantification. Peroxidation of unsaturated fatty acids in membrane phospholipids is one of the multiple cytotoxic effects of oxidative stress, and lipid peroxidation is the hallmark of toxicant-induced cellular damage. LPS-treatment induced a more than 3-fold increase in plasma lipid peroxidation levels and a similar, but smaller, increase in testicular lipid peroxidation levels at 6 h after LPS treatment. Both were attenuated by IMD cotreatment (FIG.5). The lipid peroxidation levels, in both the plasma and testes, returned to basal levels at 12 h and 72 h after LPS treatment (FIG.5).

### Effect of IMD on LPS-induced Proinflammatory Cytokines Expression in the Testes

**Figure 6:**
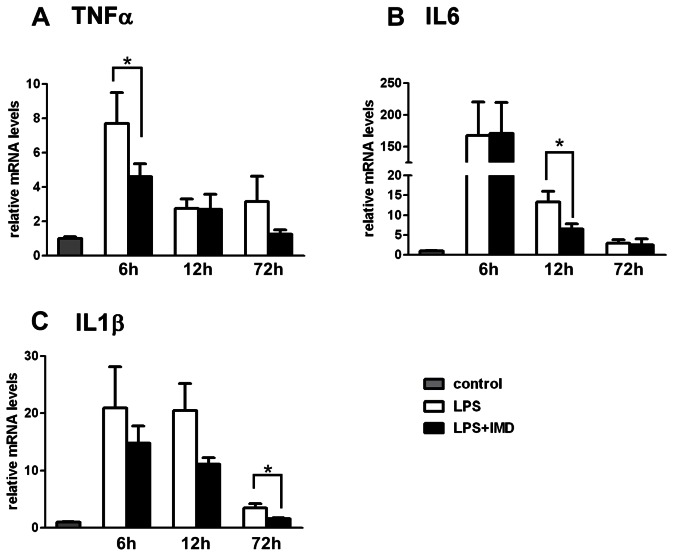
Effects of IMD cotreatment on the LPS-induced gene expression of proinflammatory cytokines in rat testis. The relative gene expression of TNFα, IL6 and IL1β in the testes 6 h, 12 h, 72 h after LPS treatment or LPS and IMD cotreatment compared with saline control was analyzed by QPCR. (A) Cotreatment of IMD suppressed the LPS-induced increase in TNFα expression at 6 h after treatment. (B) Cotreatment of IMD suppressed the LPS-induced increase in IL6 expression at 12 h after treatment. (C) Cotreatment of IMD suppressed the LPS-induced increase in IL1β expression at 72 h after treatment. Beta-actin served as the reference gene; all data were expressed as mean ± SEM; * P<0.05 by one-way ANOVA followed by Student–Newmann–Keuls test; n = 5–7.

LPS-treatment induced a dramatic increase in the testicular proinflammatory cytokine TNFα, IL6 and IL1β expression, which peaked at 6 h after treatment, and maintained a relatively high level in both the LPS alone and IMD cotreatment groups at 12 h and 72 h after LPS treatment compared with the saline control. Cotreatment of IMD attenuated but did not eliminate TNFα induction at 6 h, IL6 at 12 h and IL1β at 72 h after treatment (FIG.6).

### Effect of LPS on Testicular Expression of IMD and its Receptor Components

**Figure 7:**
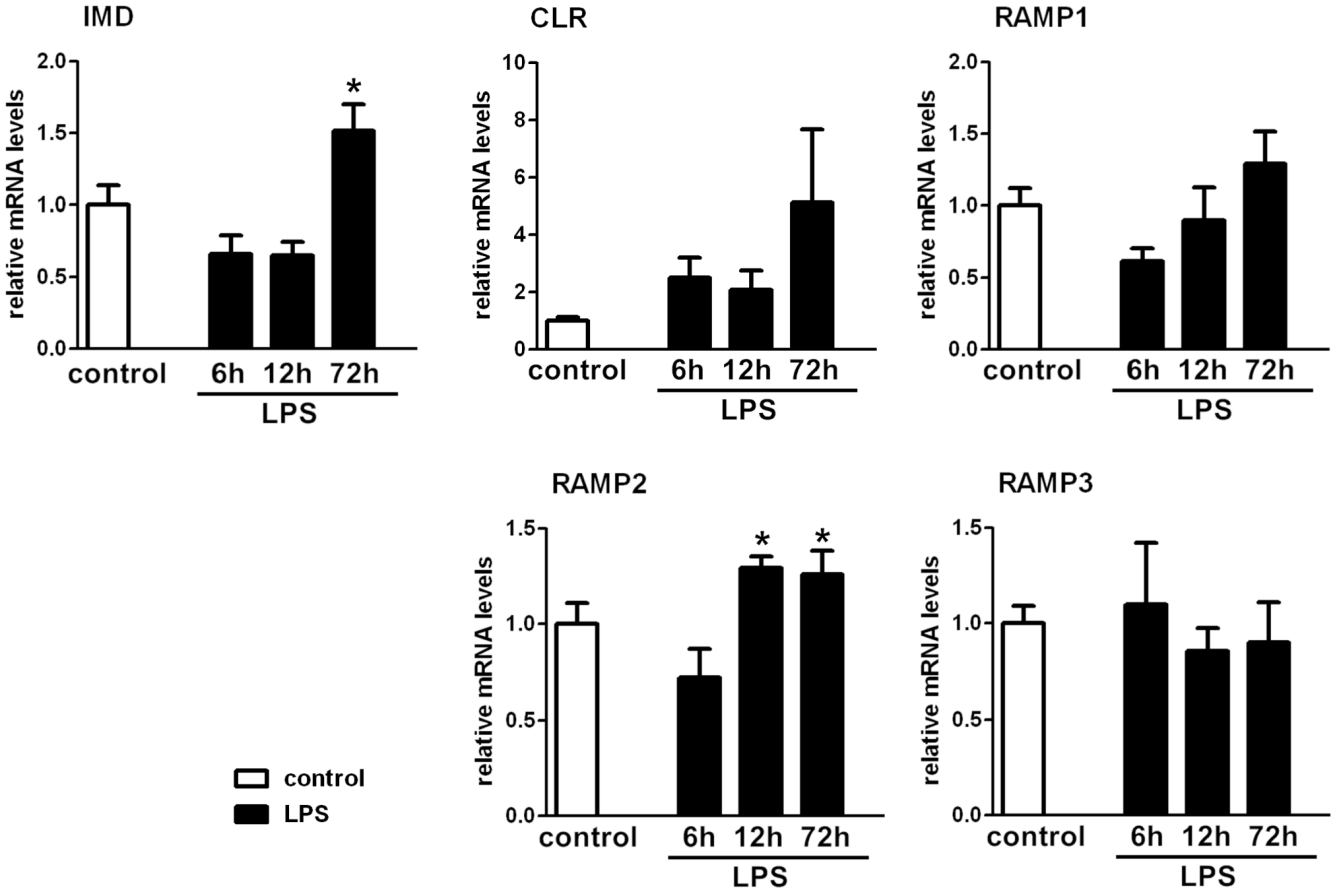
Effects of LPS on the gene expression of IMD and its receptor components in rat testis. The relative gene expression of IMD and its receptors components CLR, RAMP1, RAMP2 and RAMP3 in the testis at 6 h, 12 h, 72 h after LPS treatment was analyzed by QPCR. LPS treatment showed an increased expression of IMD and RAMP2 in rat testis. Beta-actin served as the reference gene; all data were expressed as mean ± SEM; * P<0.05 by one-way ANOVA followed by Student–Newmann–Keuls test; n = 5–7.

The effect of LPS-treatment on IMD and its receptor components’ testicular gene expression was evaluated by QPCR. LPS treatment resulted in an increase in testicular IMD and RAMP2 expression. The IMD expression level increased at 72 h after LPS treatment while the RAMP2 expression level increased at 12 h and 72 h after LPS treatment (FIG.7).

## Discussion

High expression levels of both IMD and its receptor complex components CLR/RAMPs were detected in rat testes in our study (FIG.1). The expression of CLR/RAMPs was previously reported in rat testes [18]. IMD RNA was not detected by Takei et al. in mouse testes [7]. The discrepancy may be due to differences in species (rat vs. mouse) and detection methods (QPCR vs. RT-PCR). We are the first group to report the protein localization of IMD (FIG.2) and its high levels in the testes (Table 1). The high level of IMD and the presence of its receptor components in the testes are consistent with its biological role(s) of protecting against the impairment of steroidogenesis and spermatogenesis by LPS treatment.

Testes can be compartmentalized functionally and anatomically into the gamete and endocrine compartments where spermatogenesis and steroidogenesis take place respectively. Consistent with the results from previous studies, LPS treatment in this study compromised testicular function at multiple levels, including decreased steroidogenesis and impaired spermatogenesis [4], [20]. IMD showed protective effects against both types of LPS-induced damage. IMD treatment did not prevent the decrease in testosterone production at 6 h and 12 h after LPS treatment, although it eliminated the increase in ROS levels at 6 h after LPS injection, and greatly attenuated the increase in the gene expression of TNFα at 6 h, of IL6 at 12 h and IL1β at 72 h after LPS injection (FIG.3, FIG.6.). It is important to note here that infusion of IMD for 10 min was sufficient to prevent the increase in ROS and cytokine expression 6 h to 72 h after LPS injection, and restored the plasma testosterone level to normal at 72 h after LPS injection. The restorative effect of IMD on LPS-suppressed testosterone secretion might be due to direct effects on both the testicular ROS production and the proinflammatory cytokines (TNFα, IL6 and IL1β) production. ROS are rapidly released from the activated immune system [21] after LPS injection, probably from the interstitial macrophages [22] and spermatozoa [23]. The suppression of ROS by IMD treatment to basal level at 6 h was not accompanied by the restoration of plasma testosterone levels, which indicates that ROS suppression alone could not reverse the LPS-suppressed testosterone production in vivo (FIG.5). However, the in vitro finding of a role of IMD in preventing the decrease in testosterone production by hydrogen peroxide (an ROS) in primary Leydig cells is in agreement with the in vivo data on the IMD effect on ROS (FIG.3C). The prolonged suppression of testosterone levels up to 12 h post-treatment might be due to the proinflammatory cytokines TNFα and IL6 since their expression levels were only attenuated and not eliminated at 6 h (TNFα) and 12 h (IL6) after LPS treatment while they were restored at 72 h (TNFα, IL6 and IL1β) after IMD co-treatment. Previous reports showed in testis, the proinflammatory cytokines including IL1β from interstitial macrophages [24], IL6 from interstitial macrophages [25], Leydig cells [26], Sertoli cells [27] and TNFα from macrophages [28] and spermatocytes [29] all inhibited testosterone production by Leydig cells [30].

On the other hand, IMD co-treatment did restore the impaired spermatogenesis, with effects including the accumulation of immature germ cells in the seminiferous tubule lumen at 6 h post-LPS treatment, and the increased inter-cellular gaps in the seminiferous epithelium and accumulation of round immature germ cells in the epididymal lumen at 72 h post-LPS treatment (FIG.4). IMD prevented the accumulation of immature germ cells in the seminiferous tubule at 6 h post treatment, before the attenuation of IMD on IL6 and IL1β expression. Presumably this was due to the effect of IMD in preventing the increase in ROS and was also independent of the decrease in testosterone production although testosterone is known to play a very important role in spermatogenesis.

The increased gene expression of IMD and RAMP2 (FIG.7) in the testes after LPS treatment implies that IMD may act partially in an autocrine or paracrine manner in the testes via binding to the CLR/RAMP2 receptor system. Although the study on the effect of LPS on the gene expression of IMD indicates that there was a significant increase in IMD expression at 72 h after LPS injection, the return of the plasma testosterone levels to normal was not observed in the LPS treated group without exogenous IMD co-treatment, suggesting this restoration at 72 h was not solely due to the increase in testicular IMD expression.

The localization of immunoreactive IMD both in the interstitial Leydig cells and in the spermatids inside the seminiferous tubules was consistent with its known effects of targeting different cell types in the testes (FIG.2). At present we know that the action of IMD is mediated by CLR/RAMPs, in which RAMP1–3 acts as molecular chaperones for transporting CLR from the endoplasmic reticulum and Golgi apparatus to the cell surface [31]. The downstream signaling pathways of CLR include the PI3K/Akt, cAMP/PKA and MAPK signaling cascade [31]. To fully understand the detailed mechanism, further investigation is required on which specific IMD receptor(s) and downstream signaling pathways IMD functions through.

Altogether IMD, through the suppression of ROS production and the proinflammatory cytokines expression, attenuated the LPS-induced suppression of steroidogenesis in Leydig cells and the damage to spermatogenesis. Our results suggest important pathophysiological roles for IMD in the testes for its intrinsic antioxidant capacity and regulation of proinflammatory cytokines production, implying the therapeutic potential of IMD in pathological conditions such as orchitis.

## Acknowledgments

We acknowledge the assistance of Miss Rachel Corbett in improving the English grammar, particularly syntax of the manuscript.

## References

[1] McLachlan RI, de Kretser DM (2001) Male infertility: the case for continued research. Med J Aust 174: 116–117. 11247612 10.5694/j.1326-5377.2001.tb143180.x

[2] Tremellen K (2008) Oxidative stress and male infertility–a clinical perspective. Hum Reprod Update 14: 243–258. 18281241 10.1093/humupd/dmn004

[3] Turner TT, Lysiak JJ (2008) Oxidative stress: a common factor in testicular dysfunction. J Androl 29: 488–498. 18567643 10.2164/jandrol.108.005132

[4] Reddy MM, Mahipal SV, Subhashini J, Reddy MC, Roy KR, et al (2006) Bacterial lipopolysaccharide-induced oxidative stress in the impairment of steroidogenesis and spermatogenesis in rats. Reprod Toxicol 22: 493–500. 16644180 10.1016/j.reprotox.2006.03.003

[5] Allen JA, Diemer T, Janus P, Hales KH, Hales DB (2004) Bacterial endotoxin lipopolysaccharide and reactive oxygen species inhibit Leydig cell steroidogenesis via perturbation of mitochondria. Endocrine 25: 265–275. 15758255 10.1385/ENDO:25:3:265

[6] Roh J, Chang CL, Bhalla A, Klein C, Hsu SY (2004) Intermedin is a calcitonin/calcitonin gene-related peptide family peptide acting through the calcitonin receptor-like receptor/receptor activity-modifying protein receptor complexes. J Biol Chem 279: 7264–7274. 14615490 10.1074/jbc.M305332200

[7] Takei Y, Inoue K, Ogoshi M, Kawahara T, Bannai H, et al (2004) Identification of novel adrenomedullin in mammals: a potent cardiovascular and renal regulator. FEBS Lett 556: 53–58. 14706825 10.1016/s0014-5793(03)01368-1

[8] Taylor MM, Bagley SL, Samson WK (2005) Intermedin/adrenomedullin-2 acts within central nervous system to elevate blood pressure and inhibit food and water intake. Am J Physiol Regul Integr Comp Physiol 288: R919–927. 15576658 10.1152/ajpregu.00744.2004

[9] Chen L, Kis B, Hashimoto H, Busija DW, Takei Y, et al (2006) Adrenomedullin 2 protects rat cerebral endothelial cells from oxidative damage in vitro. Brain Res 1086: 42–49. 16616051 10.1016/j.brainres.2006.02.128

[10] Muller-Redetzky HC, Kummer W, Pfeil U, Hellwig K, Will D, et al (2012) Intermedin stabilized endothelial barrier function and attenuated ventilator-induced lung injury in mice. PLoS One 7: e35832. 22563471 10.1371/journal.pone.0035832 PMC3341380

[11] Bell D, McDermott BJ (2008) Intermedin (adrenomedullin-2): a novel counter-regulatory peptide in the cardiovascular and renal systems. Br J Pharmacol 153 Suppl 1S247–262. 17965749 10.1038/sj.bjp.0707494 PMC2268039

[12] Zhao L, Peng DQ, Zhang J, Song JQ, Teng X, et al (2012) Extracellular signal-regulated kinase 1/2 activation is involved in intermedin1–53 attenuating myocardial oxidative stress injury induced by ischemia/reperfusion. Peptides 33: 329–335. 22244813 10.1016/j.peptides.2011.12.016

[13] Hagiwara M, Bledsoe G, Yang ZR, Smith RS Jr, Chao L, et al (2008) Intermedin ameliorates vascular and renal injury by inhibition of oxidative stress. Am J Physiol Renal Physiol 295: F1735–1743. 18829738 10.1152/ajprenal.90427.2008 PMC2604821

[14] Chauhan M, Balakrishnan M, Yallampalli U, Endsley J, Hankins GD, et al (2011) Adrenomedullin 2/intermedin regulates HLA-G in human trophoblasts. Biol Reprod 85: 1232–1239. 21816853 10.1095/biolreprod.110.086835 PMC3223254

[15] Chauhan M, Yallampalli U, Dong YL, Hankins GD, Yallampalli C (2009) Expression of adrenomedullin 2 (ADM2)/intermedin (IMD) in human placenta: role in trophoblast invasion and migration. Biol Reprod 81: 777–783. 19535789 10.1095/biolreprod.108.074419 PMC2754890

[16] Chauhan M, Elkins R, Balakrishnan M, Yallampalli C (2011) Potential role of intermedin/adrenomedullin 2 in early embryonic development in rats. Regul Pept 170: 65–71. 21640761 10.1016/j.regpep.2011.05.011 PMC3132565

[17] Havemann D, Balakrishnan M, Borahay M, Theiler R, Jennings K, et al.. (2013) Intermedin/Adrenomedullin 2 Is Associated With Implantation and Placentation via Trophoblast Invasion in Human Pregnancy. J Clin Endocrinol Metab. 10.1210/jc.2012-2172 PMC3565110 23337723

[18] Chang CL, Wang HS, Soong YK, Huang SY, Pai SY, et al (2011) Regulation of oocyte and cumulus cell interactions by intermedin/adrenomedullin 2. J Biol Chem 286: 43193–43203. 22009752 10.1074/jbc.M111.297358 PMC3234849

[19] Li L, Wong CK (2008) Effects of dexamethasone and dibutyryl cAMP on stanniocalcin-1 mRNA expression in rat primary Sertoli and Leydig cells. Mol Cell Endocrinol 283: 96–103. 18187254 10.1016/j.mce.2007.11.028

[20] O’Bryan MK, Schlatt S, Phillips DJ, de Kretser DM, Hedger MP (2000) Bacterial lipopolysaccharide-induced inflammation compromises testicular function at multiple levels in vivo. Endocrinology 141: 238–246. 10614644 10.1210/endo.141.1.7240

[21] Victor VM, De la Fuente M (2003) Several functions of immune cells in mice changed by oxidative stress caused by endotoxin. Physiol Res 52: 789–796. 14640902

[22] Wei RQ, Yee JB, Straus DC, Hutson JC (1988) Bactericidal activity of testicular macrophages. Biol Reprod 38: 830–835. 2840982 10.1095/biolreprod38.4.830

[23] Aitken RJ, Clarkson JS (1987) Cellular basis of defective sperm function and its association with the genesis of reactive oxygen species by human spermatozoa. J Reprod Fertil 81: 459–469. 2828610 10.1530/jrf.0.0810459

[24] Stephan JP, Syed V, Jegou B (1997) Regulation of Sertoli cell IL-1 and IL-6 production in vitro. Mol Cell Endocrinol 134: 109–118. 9426154 10.1016/s0303-7207(97)00172-x

[25] Kern S, Robertson SA, Mau VJ, Maddocks S (1995) Cytokine secretion by macrophages in the rat testis. Biol Reprod 53: 1407–1416. 8562698 10.1095/biolreprod53.6.1407

[26] Boockfor FR, Wang D, Lin T, Nagpal ML, Spangelo BL (1994) Interleukin-6 secretion from rat Leydig cells in culture. Endocrinology 134: 2150–2155. 8156916 10.1210/endo.134.5.8156916

[27] Cudicini C, Kercret H, Touzalin AM, Ballet F, Jegou B (1997) Vectorial production of interleukin 1 and interleukin 6 by rat Sertoli cells cultured in a dual culture compartment system. Endocrinology 138: 2863–2870. 9202229 10.1210/endo.138.7.5289

[28] Bryniarski K, Szczepanik M, Ptak M, Ptak W (2005) The influence of collagenase treatment on the production of TNF-alpha, IL-6 and IL-10 by testicular macrophages. J Immunol Methods 301: 186–189. 15982664 10.1016/j.jim.2005.04.002

[29] De SK, Chen HL, Pace JL, Hunt JS, Terranova PF, et al (1993) Expression of tumor necrosis factor-alpha in mouse spermatogenic cells. Endocrinology 133: 389–396. 8319585 10.1210/endo.133.1.8319585

[30] Guazzone VA, Jacobo P, Theas MS, Lustig L (2009) Cytokines and chemokines in testicular inflammation: A brief review. Microsc Res Tech 72: 620–628. 19263422 10.1002/jemt.20704

[31] Kuwasako K, Cao YN, Nagoshi Y, Tsuruda T, Kitamura K, et al (2004) Characterization of the human calcitonin gene-related peptide receptor subtypes associated with receptor activity-modifying proteins. Mol Pharmacol 65: 207–213. 14722252 10.1124/mol.65.1.207

